# Intra-host viral population dynamics during acute hepatitis E virus infection

**DOI:** 10.1128/mbio.01151-26

**Published:** 2026-07-10

**Authors:** Saskia Janshoff, Ricarda Plümers, Alina Kohl, Maximilian Klaus Nocke, Patrick Behrendt, Cornelius Knabbe, Rui Costa, Tanja Vollmer, Daniel Todt, Eike Steinmann, Andre Gömer

**Affiliations:** 1 Department of Molecular and Medical Virology, Ruhr University Bochum 9142, Bochum, Germany; 2 Department of Translational and Computational Infection Research (TRACiR), Ruhr University Bochum 9142, Bochum, Germany; 3 Institut für Laboratoriums- und Transfusionsmedizin, Herz- und Diabeteszentrum Nordrhein-Westfalen, Universitätsklinikum der Ruhr-Universität Bochum, Bad Oeynhausen, Germany; 4 Hepatitis E Virus Research Hub (HepE-Hub), Bochum, Germany; 5 European Virus Bioinformatics Center (EVBC), Jena, Germany; 6 TWINCORE, Centre for Experimental and Clinical Infection Research, a joint venture between Helmholtz Centre for Infection Research and Hannover Medical School, Hannover, Germany.; 7 Department of Gastroenterology, Hepatology, Infectious Diseases and Endocrinology, Hannover Medical School, Hannover, Germany; 8 German Center for Infection Research (DZIF), Braunschweig, Germany; 9 Institut für Laboratoriums- und Transfusionsmedizin, Herz- und Diabeteszentrum Nordrhein-Westfalen, Universität Bielefeld, Bad Oeynhausen, Germany; Ulm University Medical Center, Ulm, Baden-Württemberg, Germany

**Keywords:** hepatitis E virus (HEV), high-throughput sequencing, acute infection, intra-host evolution, blood donors

## Abstract

The hepatitis E virus (HEV) is the most common cause of acute viral hepatitis worldwide. RNA viruses like HEV can establish viral populations with high intra-host variability, enabling them to rapidly adapt to changing immune responses, as observed in chronic infections. This study aimed to investigate how intra-host evolution shapes HEV populations during acute asymptomatic infection. Using a highly sensitive HEV amplicon sequencing approach, we characterized intra-host viral diversity and mutational signatures present in serum samples from a cohort of over 80 asymptomatic blood donors infected with acute HEV-3. The overall diversity within the host was constrained; however, several recurrent substitutions were identified, and four mutations in the polymerase region were found to be enriched among the donors. Despite being markedly replication-deficient *in vitro*, their replication defects could be rescued in an RNA-dependent RNA polymerase trans-complementation assay. Longitudinal sampling in a subset of donors revealed temporal shifts in variant frequencies, indicating ongoing early selection dynamics. In contrast to chronic HEV infection in immunosuppressed patients, acute-phase populations exhibited a significantly lower number of single-nucleotide variants (SNVs) and an absence of high-frequency variants. The presence of premature stop codons and other defective genomes was primarily detected during the acute phase of infection. This finding further supports the hypothesis that the early evolutionary landscape is highly dynamic, but constrained. Although HEV diversity is markedly restricted during acute infection, early evolutionary changes indicate that selective forces act even within the short window of acute, self-limiting, and asymptomatic disease. These findings offer mechanistic insights into early intra-host evolution, highlight conditions under which deleterious variants can transiently persist, and lay the groundwork for linking genotypic features to clinical outcomes and treatment responsiveness.

**IMPORTANCE:**

The hepatitis E virus is the leading cause of acute viral hepatitis globally. While studies of chronic infections have shown that HEV can evolve and adapt in ways that influence antiviral treatment responses, little is known about how the virus changes during the early, acute phase of infection. By analyzing viral populations in asymptomatic blood donors, this study demonstrates that HEV undergoes dynamic evolutionary changes even during short, self-limiting infections. Although overall viral diversity remains restricted, selective pressures still drive the emergence of new variants, including some with impaired replication capacity. These findings provide important mechanistic insight into early intra-host viral evolution and the conditions that allow defective variants to transiently persist. This work establishes a foundation for future studies linking viral genetic variation to disease progression, clinical outcomes, and treatment responsiveness.

## INTRODUCTION

The hepatitis E virus (HEV) is a (+) single-stranded RNA virus and a leading cause of acute viral hepatitis worldwide. It is responsible for an estimated 19.47 million acute infections each year (1). In most cases, acute HEV infection is asymptomatic and resolves within a few weeks. However, in certain populations, such as individuals with pre-existing liver disease or pregnant individuals, infection can lead to severe symptoms, high viremia, and significantly increased mortality rates (2, 3). Beyond its typical acute course, HEV can establish a chronic infection (chronic hepatitis E [CHE]), primarily occurring in immunocompromised individuals such as solid organ transplant recipients, patients undergoing chemotherapy, or individuals infected with HIV (4–6). Chronic HEV infection contributes to ongoing liver inflammation and fibrosis, yet treatment options remain limited. Currently, interferon alpha or off-label ribavirin (RBV) are the only recommended treatments, while sofosbuvir has been explored as an alternative therapy but has shown limited efficacy. Crucially, they are associated with severe side effects, and their efficacy is limited in some patients. This underscores the urgent need for new therapeutic options (7).

An important consideration in the treatment of HEV infection and the progression of the disease is its capacity to evolve. HEV has diversified into at least eight distinct genotypes (HEV-1 to HEV-8), which differ in terms of their transmission routes, host range, and clinical outcomes (8). In Europe, HEV-3 is the most prevalent genotype and is subdivided into multiple subtypes (HEV-3a to HEV-3m and HEV-3ra). At the intra-host level, HEV’s evolutionary potential is particularly evident in chronically infected patients, especially under antiviral pressure. A notable instance of this is the G1634R substitution in the viral polymerase, which can emerge during RBV treatment and has been associated with enhanced viral replication capacity *in vitro* (9–11). Similarly, the A1343V substitution emerges rapidly under sofosbuvir exposure and has been associated with viral rebound, thus limiting the effect of sofosbuvir *in vivo* (12–14). These findings demonstrate HEV’s significant evolutionary adaptability, a hallmark of many RNA viruses, which enables it to adapt to selective pressures such as host immunity and antiviral drugs (15, 16). This capacity for rapid genetic change, or genetic flexibility, is crucial not only for viral survival but also for influencing disease progression. In other RNA viruses, notably the hepatitis C virus (HCV), an increase in viral genetic diversity during the early phase of acute infection has been directly linked to immune evasion and an increased likelihood of progression to chronic infection (17). These findings highlight that early viral diversification can influence the progression of the disease by enabling the virus to evade the host’s immune responses before clearance is achieved. While HEV shares features with HCV as an RNA virus with the capacity for rapid evolution, its intra-host dynamics during acute infection remain largely unexplored. Despite mounting evidence of HEV’s adaptability in chronic cases, particularly under antiviral pressure, it is unclear whether early-stage viral evolution contributes to disease severity, prolonged viremia, or eventual chronicity in a similar way. To address this question, we sequenced over 80 serum samples from blood donors with acute, asymptomatic HEV infection. Using this material, we determine the frequency of resistance-associated variants in treatment-naïve virus populations within the viral polymerase, generate variant profiles, and characterize mutational signatures from serially sampled individuals. Our result depicts marked differences in the composition of intra-host populations of HEV during acute infection versus patients with persistent infection.

## MATERIALS AND METHODS

### Blood donors and hepatitis E virus nucleic acid screening

We analyzed blood donations that were previously identified as HEV RNA-positive and collected as part of Plümers et al. (18). These samples were obtained through routine HEV screening conducted by Uni.Blutspendedienst OWL, which includes donors in North Rhine-Westphalia, Lower Saxony, and Hessen between January 2015 and December 2022. For the present study, 123 serum samples were available for follow-up molecular analysis. The HEV-positive blood donors who were serially sampled between May 2011 and January 2012 were first described in reference 19. All samples were stored at –80°C until further processing.

### RNA extraction & amplicon generation and sequencing

Total RNA was extracted from 500 µL of plasma using the AltoStar AM16 automated extraction system (Altona Diagnostics Technologies) according to the manufacturer’s protocols. Complementary DNA (cDNA) synthesis and amplicon generation were conducted in accordance with the protocol outlined in reference 11. Next-generation sequencing (NGS) libraries were prepared using the Nextera XT DNA Library Preparation Kit (Illumina, San Diego, CA, USA), after which the libraries were sequenced on either the MiSeq platform (PE250) or the NovaSeq platform (PE150).

### Intra-host diversity analysis

With few alterations, variant analysis followed the pipeline described by Gömer et al. (20). Raw sequencing data were trimmed using Trimmomatic v0.39 (21), and their quality was assessed using FastQC (22). After aligning the reads to the reference wbGer27 (FJ705359), baseline consensus sequences were generated using sam2consensus (https://github.com/edgardomortiz/sam2consensus/). The baseline serum consensus sequence of each donor was afterward used as a reference for read mapping via tanoti (https://bioinformatics.cvr.ac.uk/software/tanoti/) and subsequent variant detection. For each patient with multiple samples, the earliest obtained baseline serum consensus was used as a reference for mapping and to detect variations over time. Variant calling was performed on the polymerase region on nucleotide positions 3996–4953 within ORF1 as well as the ORF2/3 coding region on nucleotide positions 5134–6320 using DiversiTools (https://github.com/josephhughes/DiversiTools/) and vNvS tools (https://github.com/rjorton/vnvs/). For the multiple sequence alignment, ClustalW (2.1) (23) was used, followed by a phylogenetic analysis using IQ-TREE 2 (2.4.0) (24) to calculate a maximum-likelihood tree and obtain branch supports with the ultrafast bootstrap (UFBoot) (25). Sequences were genotyped according to Smith et al. (8). A home-made pipeline was developed to manage the tool execution via Python (3.12.3). Data visualization was carried out in R (4.5.0) using custom scripts and the following packages: tidyverse, ggpubr, ggplot2, and ggtree (26–28).

### Database analysis

Publicly available Paslahepevirus sequences were accessed through the NCBI Virus database on 24 March 2025. All entries assigned to the genus (Taxon ID: 2948857) were retrieved from GenBank. These entries were subsequently filtered to remove low-quality or incomplete sequences. This process yielded a final set of 1,199 full-length polymerase sequences. These sequences were aligned using MAFFT v7.450, and downstream analyses—including variant frequency estimation—were conducted in R using the Biostrings package.

### HEV replicon assays

Human hepatoma (HepG2) cells were cultured in Dulbecco’s Modified Eagle Medium (Gibco) supplemented with 10% (vol/vol) fetal bovine serum (Gibco), 100 µg/mL streptomycin, 100 IU/mL penicillin (Invitrogen), 2 mM L-glutamine, and 1% nonessential amino acids (Invitrogen). Cells were maintained at 37°C in a 5% CO_2_ incubator on culture dishes pre-coated with collagen (SERVA Electrophoresis). The subgenomic *Gaussia* luciferase reporter replicon (p6-Gluc) based on the Kernow-C1 p6 strain was used as the parental wild-type (WT) control. Patient-derived substitutions L1368F, T1472Y, G1550V, and G1597W, and the polymerase-inactive control (GAA), were introduced into the p6-Gluc backbone via site-directed mutagenesis PCR. Plasmids were linearized with MluI, purified, and used as templates for *in vitro* transcription using T7 RNA polymerase, as previously described (Todt et al. [16]). Generated RNA was purified using the NucleoSpin RNA Clean-Up Kit (Macherey & Nagel), and RNA integrity was verified by agarose gel electrophoresis. For electroporation, 5 × 10^6^ HepG2 cells were resuspended in 400 µL Cytomix and pulsed using a Gene Pulser Xcell apparatus (Bio-Rad). To assess replicative fitness (mono-electroporation), cells were electroporated with 2.5 µg of the respective variant replicon RNA mixed with 2.5 µg of tRNA. To assess trans-complementation (co-electroporation), cells were electroporated with a mix of 2.5 µg of variant replicon RNA and 2.5 µg of full-length WT KernowC1/p6 RNA. Following electroporation, cells were seeded in 96-well plates. Supernatants were collected at 4, 24, 48, and 72 hours post-electroporation. *Gaussia* luciferase activity was measured using a Centro XS3 LB 960 luminometer (Berthold Technologies) by adding coelenterazine substrate to 20 µL of supernatant. Productive replication of the full-length WT genome in the co-electroporation setup was confirmed by immunofluorescence staining for the HEV ORF2 protein using a polyclonal anti-HEV antibody.

### Software

Figures were generated using the programming language R and final figure adjustments were partly done in Adobe Illustrator.

## RESULTS

### Identification of HEV-3 subtype-specific mutations and resistance-associated variants in acutely infected blood donors

**Fig 1:**
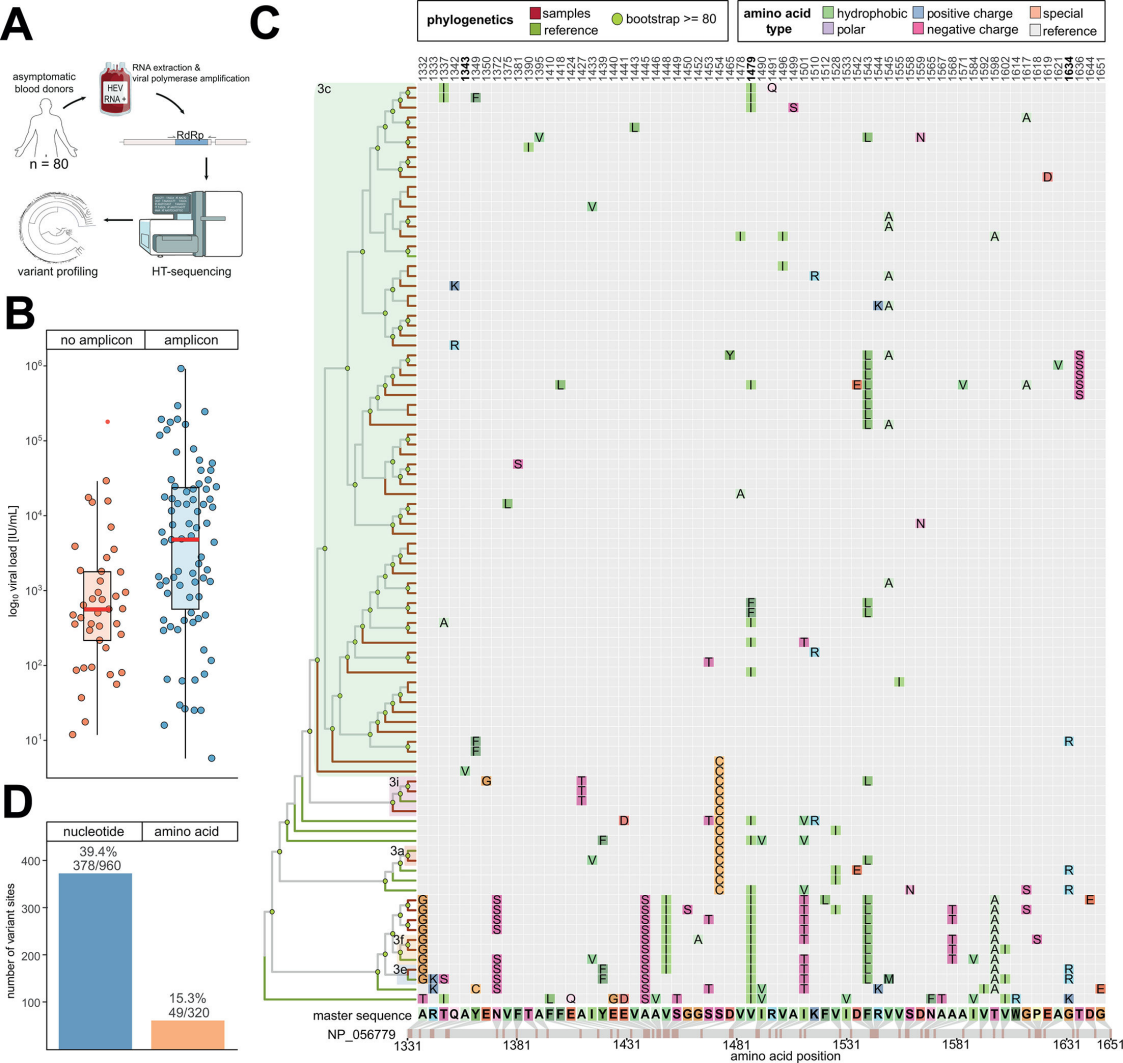
Intra-host viral load, phylogeny, and sequence diversity of HEV isolates from acutely infected blood donors. (**A**) Framework of the current study. (**B**) Viral loads (IU/mL) of HEV RNA-positive blood donors, stratified by successful (blue) versus unsuccessful (orange) amplicon generation. Red bars indicate the median, and box edges indicate the interquartile ranges. Outlier shown as a smaller dot. (**C**) Cladogram from a maximum-likelihood tree showing HEV sequences from donor samples (red tips) alongside reference sequences (green tips). Branch support values of at least 80 are marked with green circles. The highlighter plot shows the distribution of amino acid substitutions across the genome relative to the HEV-3c reference, with positions according to NP_056779. (**D**) The number and proportion of variable sites across the polymerase amplicon. The bars show nucleotide (blue) and amino acid (orange) substitutions relative to the reference. Numbers above bars indicate the number of polymorphisms and their relative frequency. Pictograms were generated using Biorender.

To investigate the evolutionary capacity of HEV during an acute infection, we identified blood donors who tested positive for HEV RNA. A total of 123 HEV RNA-positive donations were selected for analysis of viral diversity within the RNA-dependent RNA polymerase (RdRp) region (Fig. 1A). The mean viral load across all donors was 2.88 × 10^4^ IU/mL (range 5.72 × 10^0^–9.14 × 10^5^ IU/mL). PCR amplicons were successfully generated in 80 samples (65%), with amplification success showing an association with viral load (Fig. 1B). Donors with successful amplification had higher viral loads, on average 8.5-fold greater (median 4.8 × 10^3^ IU/mL, range: 5.72 × 10^0^–9.14 × 10^5^) than those for whom amplification failed (median 5.6 × 10^2^ IU/mL, range: 11.7 × 10^1^–1.75 × 10^5^). Samples yielding successful amplicons were subjected to short-read, high-throughput sequencing for viral genome characterization. Phylogenetic analysis of the resulting consensus sequences revealed a predominance of HEV genotype 3 subtype c (86.2% of sequences), followed by subtypes 3f (7.5%), 3i (3.8%), 3a (1.2%), and 3e (1.2%) (Fig. 1C and Fig. S1A). A total of 378 out of 960 nucleotide positions (39.4%) and 49 out of 320 amino acid residues (15.3%) in the RdRp region were found to exhibit variability on consensus level (Fig. 1D). Several substitutions were subtype-specific: for example, strains classified as subtype HEV-3ef shared nine substitutions that were distinct from those in other sequences. Many of these substitutions were located within the Palm domain (Fig. S1B).

Notably, substitutions that commonly emerge in patients with chronic HEV infection undergoing antiviral treatment and which were associated with treatment efficacy were also detected in donors with acute asymptomatic infection. For example, the A1343V variant was identified at consensus level in one individual (Fig. 1C). This variant emerges during sofosbuvir treatment at high frequencies and is associated with antiviral resistance (13). The V1479I/F substitution was detected in 19 donors. While its exact function is unknown, database records and previous reports suggest it to be a variable site that is associated with ribavirin treatment (29, 30). Furthermore, the G1634R substitution, which enhances replicative fitness *in vitro* and has been associated with ribavirin treatment, was identified in two blood donors (9, 11). Importantly, the detection of these substitutions in acutely infected, treatment-naïve individuals does not imply an association with chronic infection or an increased propensity for chronic disease, but rather indicates that such variants can also occur as part of the natural viral diversity circulating in patients. In summary, we sequenced the RdRp region of 80 HEV-3 isolates obtained from blood donors with acute infection. These isolates demonstrate both subtype- and isolate-specific variation in nucleotide and amino acid composition, reflecting the genetic diversity of circulating HEV strains.

### Intra-host diversity constrained in acute versus chronic infection

**Fig 2:**
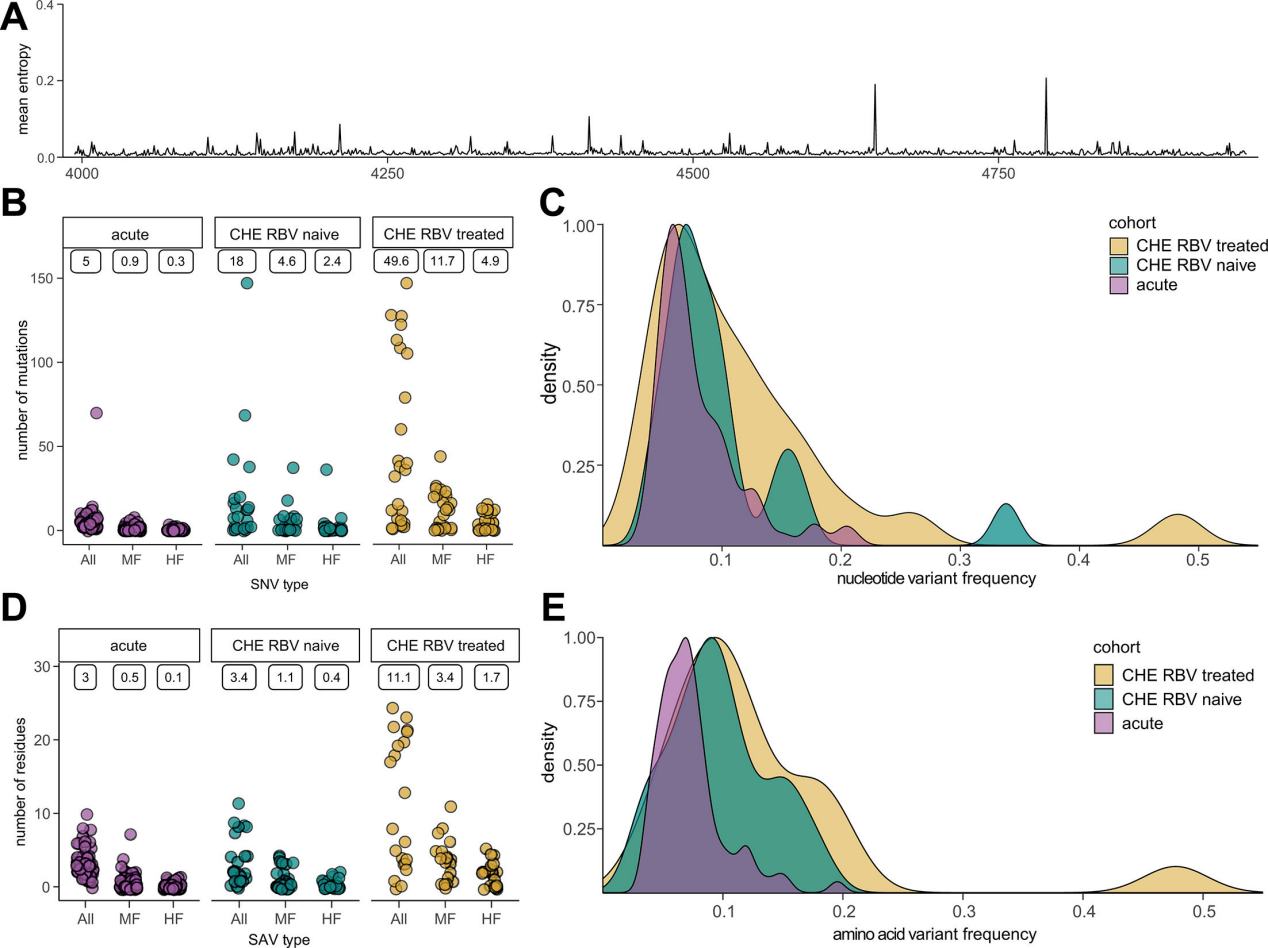
Constrained heterogeneity in donors with acute infection compared to patients with CHE. (**A**) Mean nucleotide entropy per position across all donors with acute infection included in the study (*n* = 78). (**B**) Quantification of nucleotide-level mutations for donors with acute infection (magenta), untreated CHE patients (green), and CHE patients with RBV treatment (yellow). All variants exceeding the error threshold are shown under “Al.” MF-variants represent mutations between 10% and 20% frequency, and HF-variants represent mutations above 20%. Boxes indicate the mean number of variants per patient for each variant interval. (**C**) Normalized mutation frequency density plot for the three cohorts. (**D**) Amino acid substitution across infection cohorts. Variants are categorized by frequency: all variants (above the detection threshold of 3.287%), medium-frequency variants (MF; 10%–20%), and high-frequency variants (HF; >20%). The numbers in the boxes show the mean substitution count per individual. (**E**) Variant frequency distributions across cohorts as density curves. Colors represent different patient groups: magenta (acute naïve), green (chronic naïve), and brown (chronic ribavirin-treated). The *x*-axis shows variant allele frequency, and the *y*-axis shows probability density.

To address how HEV populations were shaped during acute infection, we quantified intra-host genetic variants. To do so, we mapped the sequencing short reads from each sample to their respective consensus sequences, which enabled us to analyze viral diversity in detail without introducing subtype-specific biases into the analysis. With the exception of two samples (A32 and A70), which were excluded due to insufficient coverage, the sequencing depth was high and evenly distributed across the RdRp region (Fig. S2 and S3). The mean sequencing depth was 54394 per position (sd: ±72938), which allowed for the robust detection of both low- and high-frequency variants. To differentiate true biological variants from sequencing artifacts, we applied a conservative variant frequency threshold of 3.287% for single-nucleotide variants (SNVs) based on previously reported Illumina short-read sequencing error rates (31). All variants above this threshold were classified as true variants with further classification into low-frequency (LF-SNVs, 3.287% to <10%), medium-frequency (MF-SNVs, >10% to 20%) or high-frequency (HF-SNVs, >20%) variants. SNVs were relatively evenly distributed across the RdRp region, with most samples exhibiting predominantly LF-variants (82% of all variants were low LF-SNVs) and low Shannon entropy values (Fig. S4 through S6). When calculating the mean Shannon entropy across all donors, only a few sites showed elevated values (Fig. 2A). On average, each asymptomatic donor harbored 5 SNVs in total, including 0.9 MF-SNVs and 0.3 HF-SNVs (Fig. 2B). Notably, one donor (A6) showed signs of potential co-infection or the presence of two closely related viral populations, with 68 LF-SNVs detected across the amplicon at comparable frequencies (Fig. S4 and S5). To contextualize the intra-host variability observed at a single time point during acute HEV infection, we analyzed previously published data from immunocompromised CHE patients, including both RBV-naïve individuals and those who received RBV treatment (11). Compared to acutely infected individuals, both RBV-naïve and RBV-treated CHE patients exhibited higher overall viral diversity, as reflected by increased Shannon entropy and a greater number of SNVs (Fig. 2B and Fig. S7). Specifically, we observed a 3.4-fold increase in total SNVs in RBV-naïve patients and a 99-fold increase in RBV-treated patients (Fig. 2B). This difference was also pronounced when focusing on intermediate- and high-frequency SNVs, which were up to 16-fold more abundant in CHE patients than in acutely infected blood donors. Similarly, the SNV frequency distribution in CHE patients was markedly shifted toward higher variant frequencies (Fig. 2C). In these individuals, viral populations exhibited prominent peaks around frequencies of 0.3 and 0.5, indicating the presence of dominant variants. By contrast, acutely infected donors showed a sharp peak centered around a frequency of 0.1, suggesting a less diverse and more homogeneous viral population.

Next, our aim was to quantify amino acid substitutions to gain deeper insight into the selective pressures acting on viral populations. Overall, the RdRp region appeared to be highly conserved, with a low number of amino acid changes observed (Fig. 2D and E and Fig. S8).

On average, donors with acute infection had 3.0 substitutions above the error threshold, while patients with chronic infection had 3.4 substitutions on average without treatment and 11.1 with treatment. The difference in diversity was more pronounced when frequency intervals were considered: CHE patients without treatment had almost twice as many MF variants and four times more HF variants than blood donors with acute infection. CHE patients who received treatment showed the highest number of MF variants (3.4) and HF variants (1.7) (Fig. 2D). We then depicted the differences in frequency distribution of amino acid substitutions (Fig. 2E). Here, CHE patients who received treatment showed a distinct signal at higher frequency in the density curves, with variants clustering around a frequency of 50%, suggesting higher viral diversity and potential selection of treatment-associated variants.

In addition to the RdRp sequences, we also sequenced a fragment covering the overlapping region of ORF2 and ORF3 to investigate whether similar patterns of diversity occur in other parts of the viral genome. Although the overall amplification success rate for this region was lower compared to the RdRp, amplicons from 13 donors were generated (Fig. S9 and S10). Consistent with the intra-host diversity measured in the RdRp region of matching samples, we identified multiple variants throughout these coding regions. On average, ORF2 harbored 2.2 total variants per sample, including 0.7 MF and 0.2 HF variants. Correspondingly, ORF3 exhibited an average of 1.2 total variants, comprising 0.5 MF and 0.2 HF variants. Similar to the RdRp, fewer non-synonymous substitutions were identified than synonymous substitutions.

In summary, we quantified the viral diversity of donors with acute HEV infection and compared it to that of patients with chronic HEV infection. We found that HEV from acutely infected donors harbored fewer nucleotide and amino acid variants, most of which occurred at low frequencies, than HEV from chronically infected patients.

### Identification of a common mutational pattern

**Fig 3:**
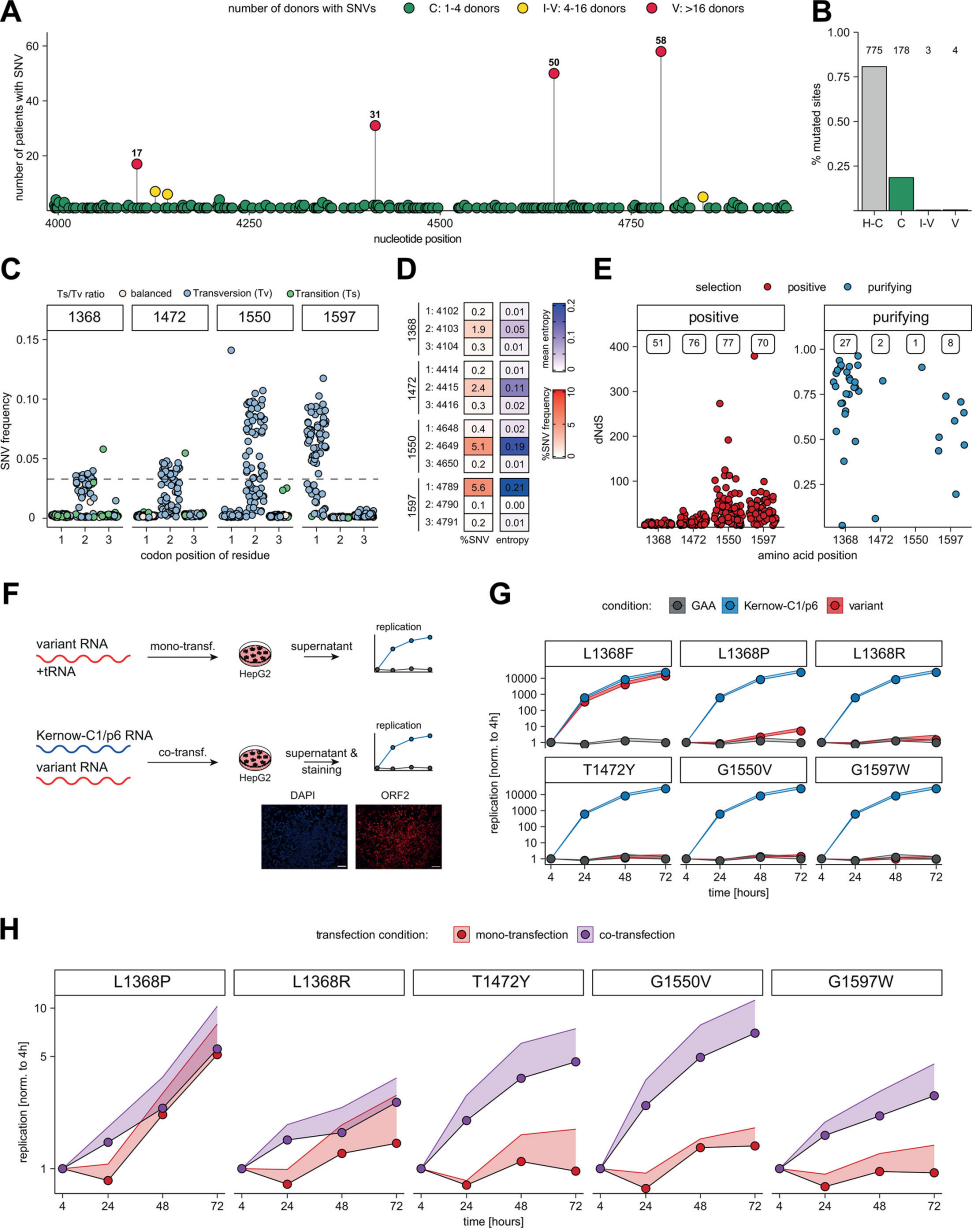
(**A**) Distribution and replicative fitness of variable nucleotide positions across 78 acutely infected donors. Sites are color-coded according to the number of donors with detectable SNVs: green (one to four donors), yellow (four to 16 donors), and red (>16 donors). Highly conserved sites without mutations have been excluded from the visualization. (**B**) Quantification of site conservation categories across the amplicon. Highly conserved sites (H-C; gray) show no detectable mutations in any of the 78 donors analyzed. Numbers indicate the count of sites in each category. (**C**) Codon position analysis for the four most variable sites (mutated in ≥16 donors). Points are colored according to the transition/transversion (Ts/Tv) ratio: green for Ts/Tv ≥ 1.1 (transition-biased), blue for Ts/Tv ≤ 0.9 (transversion-biased), and gray for 0.9 < Ts/Tv < 1.1 (balanced). (**D**) Mutational characteristics of hotspot positions, showing the mean SNV frequency and Shannon entropy values for the four most variable sites (residues 1368, 1472, 1550, and 1597). (**E**) Selection pressure analysis at hotspot positions. The left panel shows sites under positive selection (dN/dS > 1.1), and the right panel shows sites under purifying selection (dN/dS < 0.9). Numbers in boxes indicate the number of donors exhibiting each selection pattern at the specified residue positions. (**F**) Experimental setup to assess the replication competence of emerging variants. All scale bars = 100 µm. (**G**) Replicative capacity of enriched variants. Each variant was introduced into a subgenomic Gaussia luciferase replicon, and replication kinetics were monitored for 72 hours. Raw relative light units (RLU) were normalized to the 4-hour input control. Colors denote variant replicons (red) or controls (replication-deficient GAA control, gray; Kernow-C1/p6 wild-type control, blue). Shaded ribbons represent the standard deviation of three independent experiments. (**H**) Trans-complementation assay. Variant replicon RNA was co-electroporated with full-length, replication-competent Kernow-C1/p6 wild-type RNA to evaluate whether defective variants could be rescued in trans.

To identify regions with high variation across multiple patients, we categorized each nucleotide position within the RdRp amplicon based on the number of donors with detectable SNVs above 3.287%. We further classified these sites into four categories. Highly conserved (H-C) sites, which showed no SNVs above the error threshold in any donor. Conserved (C) sites with SNVs in fewer than 5% of donors. Intermediate variable (I-V) sites that had SNVs in fewer than 20% of donors. Variable (V) sites which exhibited SNVs in more than 20% of donors at the same position (Fig. 3A). Most nucleotides within the amplicon were highly conserved with no detectable mutations. In fact, 775 sites representing 86% of the amplicon did not show mutations above the error level among donors with acute infection (Fig. 3B). Although there was low overall heterogeneity in acute infections, we observed variation above the error threshold at four nucleotide positions: 4103, 4415, 4649, and 4790 (Fig. 3 and red dots). These positions were observed in at least 17 donors, suggesting that they may play a specific role in acute HEV infection. These positions correspond to amino acid residues 1368, 1472, 1550, and 1597, respectively (Fig. 3C and D). To assess how selection acts on these positions, we examined their codon location and mutational context (Fig. 3C and D). All four nucleotide positions were located in the first or second codon positions, exhibiting elevated entropy and average SNV frequencies (entropy range: 0.05–0.2; average SNV range: 1.9%–5.9%; Fig. 3D). Variants at these positions were predominantly transversions rather than transitions. Further analysis of synonymous versus non-synonymous substitutions revealed evidence of positive selection at positions 4649 (part of residue 1550) and 4790 (part of residue 1597) in most donors, whereas positions 1368 and 1472 displayed signals of negative selection (Fig. 3E). Apart from the residues under positive selection at positions 1550 and 1597, no common substitutions were observed among donors with acute infection. Subsequently, we assessed the frequency of these substitutions among 1199 HEV sequences downloaded from NCBI (Table 1). All of these were highly conserved, encoding for the consensus amino acid rather than the mutant variant. Notably, the substitution at position 1550V is located within the glycine residue of the polymerase catalytic GDD motif, which could potentially impair its function. The location of all substitutions was visualized on a 3D alpha fold prediction of the polymerase (Fig. S11). Additionally, these substitutions were absent in CHE patients, regardless of their treatment status. To investigate the functional consequences of these variants, we employed the Kernow-C1/p6 reverse genetic replicon system (Fig. 3F). Each observed substitution at residues 1368, 1472, 1550, and 1597 was introduced into the polymerase region of the KernowC1/p6 replicon, and following *in vitro* transcription and electroporation into permissive cells, replication competence was monitored in a longitudinal time-course experiment using *Gaussia* luciferase as a reporter (Fig. 3G). Except for the L1368F construct, which replicated comparably to the p6 control, all variants exhibited markedly reduced RNA replicative fitness. Substitutions at residues 1472, 1550, and 1597 were particularly deleterious, producing replication signals indistinguishable from the inactive GAA control. Given the prevalence of these variants *in vivo*, we next examined whether their replication defects could be rescued by trans-complementation with a functional polymerase (Fig. 3F). To this end, variant replicon RNAs were co-electroporated with full-length KernowC1/p6 RNA. In this dual-electroporation setup, *Gaussia* luciferase readout corresponds to translation from the variant replicon RNA, whereas ORF2 immunostaining denotes productive translation of the wild-type RNA. Using this complementation system, we observed that the T1472Y, G1550V, and G1597W variants could be trans-complemented, manifesting a threefold to sevenfold enhancement of RNA replication in the co-electroporation condition compared with mono-electroporation of the variant alone (Fig. 3H).

**TABLE 1:** Frequency of selected mutations in acutely infected individuals and in database records

Nucleotide position	AA residue	Frequency in acute	Selected above 3%	Frequency in DB
4103	L1368	98.8	78	99.7
	F1368	<1	0	<1
	P1368	<1	0	<1
	R1368	<1	0	0
4415	T1472	97.6	78	99.6
	Y1472	2.3	31	0
4649	G1550	94.6	78	100
	V1550	5	50	0
4790	G1597	94.3	78	99.8
	W1597	5.5	58	<1

To investigate potential evidence of lethal mutagenesis during HEV infection, we analyzed the cumulative counts of stop codons across treatment groups (Fig. S12). Although RBV treatment resulted in a slight increase in the number of stop codons compared to untreated CHE patients, overall accumulation remained limited in both CHE groups. In contrast, donors with acute infection exhibited substantially higher cumulative stop codon accumulation, suggesting an increased mutational burden during the acute phase of infection.

In summary, our analysis demonstrates that HEV populations are predominantly shaped by strong purifying selection, with the majority of sites remaining highly conserved across individuals. Nevertheless, we identified four nucleotide positions that were recurrently mutated in donors with acute infection and exhibited signatures of positive selection. The subsequent functional characterization of these substitutions indicated that, despite their enrichment *in vivo*, three of the four variants exhibited a substantial impairment in replicative capacity in cell culture. However, their replication defects could be restored through trans-complementation with a replication-competent parental virus, suggesting that these mutations may arise and persist transiently within heterogeneous viral populations supported by cooperative interactions during acute infection.

### Temporal mutational signatures during acute HEV infection reveal dynamic intra-host variation

**Fig 4:**
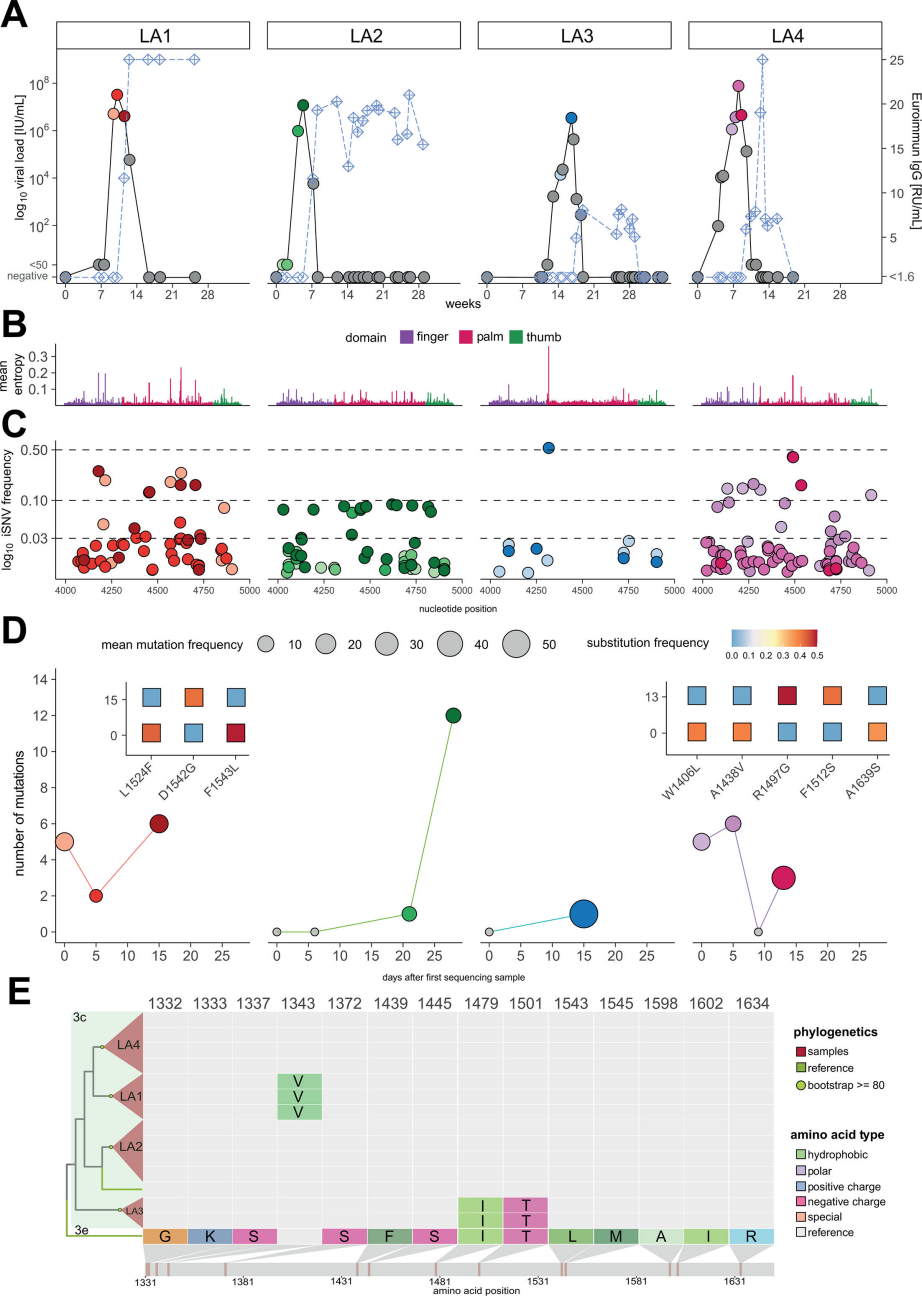
Infection and evolutionary kinetics in donors with acute HEV infection. (**A**) Temporal dynamics of HEV infection and host seroconversion in four donors (LA1–LA4). Viral load (solid black line, left *y*-axis; log_10_ IU/mL) was quantified by RT-qPCR, while anti-HEV IgG levels (dashed blue line, right *y*-axis; RU/mL) were measured by Euroimmun ELISA. Each point represents a longitudinal sample; color-coded points denote samples subjected to deep sequencing, whereas gray points indicate low viral load or samples not selected for sequencing. (**B**) Mean entropy measurements across all time points, separated by polymerase domains: fingers (purple), palm (pink), and thumb (green). (**C**) Overview of variant frequencies for each patient and time point. Time points are color-coded as in panel **A**, and dashed lines indicate error thresholds at 3.287% as well as cut-offs at 10% and 50%. (**D**) Quantification of variants exceeding the error threshold at each time point. Dot size reflects the mean frequency of detected variants. The inlets show amino acid substitutions observed at a frequency of >10% across all time points. (**E**) Consensus variant profiles for each patient at each time point.

To assess the dynamics of the intra-host diversity during the early stages of acute HEV infection, we sequenced four blood donors who had acquired HEV and provided multiple blood donations during viremia (19). This enabled us to track viral kinetics and intra-host evolution over time (Fig. 4). All four donors exhibited a rapid increase in viral loads during the first few days of sampling, rising up to 3.26 × 10^7^ IU/mL (Fig. 4A). Following the peak, viral titers declined, coinciding with increasing anti-HEV IgM and IgG responses. To assess viral evolution, we performed amplicon-based sequencing of the RdRp region at all available time points, resulting in two to four sequencing samples per donor (color-highlighted dots in Fig. 4A). For this analysis, consensus sequences from the first available time point for each patient were used as mapping references to analyze intra-host dynamics over time. Variants exceeding the error threshold were detected in all four donors, although their number and frequency varied (Fig. 4B through D). Donor LA1 had three time points sequenced, with two to six variants detected at each time point that were distributed throughout the amplicon. Some of these variants reached frequencies of above 20%, also reflected by elevated entropy at these positions (Fig. 4A through D; column 1). Donor LA2 exhibited a distinct pattern: only one variant was identified in the initial three time points. However, at the final sampling point, when the viral load peaked at 9.6 × 10^5^ IU/mL, 12 variants emerged at a similar frequency of about 10% in the viral population (Fig. 4A through D; column 2). For donor LA3, only two time points were available. No variants exceeded the threshold at the first time point, while at the second time point (15 days later), a single HF variant emerged at 53% when viral loads peaked (Fig. 4A through D; column 3). Donor LA4 had four samples sequenced. Variants were present in the first two time points, followed by a marked drop in diversity at the third time point, which corresponded with peak viremia. Subsequently, two new variants emerged at the final time point at above 10% frequency (Fig. 4A through D; column 4). We then proceeded to analyze amino acid-level changes to better understand selection processes. While in all donors nucleotide mutations were present, substitutions were only detected in donors LA1 and LA4. Donor LA1 accumulated variants at three residues (L1524F, D1542G, and F1543L), and donor LA4 at five residues (W1406L, A1438V, R1497G, F1512S, and A1639S). Consensus variant profiling of all donors showed that all four HEV isolates were from subtype 3c and that donor LA-1 had the sofosbuvir resistance-associated V1343 substitution encoded at the consensus level (Fig. 4E).

In conclusion, analyzing virus populations from serially sampled donors with acute HEV infection allowed us to monitor the emergence of nucleotide and amino acid variants within short time frames. These longitudinal data emphasize the dynamic nature of HEV evolution during acute infection. This is characterized by changing viral diversity, transient variant emergence, and donor-specific patterns of intra-host selection. While some individuals exhibited stable viral populations, others experienced more rapid changes in variant composition.

## DISCUSSION

In this study, we used a highly sensitive HEV amplicon sequencing approach to analyze mutational signatures and intra-host viral diversity in over 80 blood donors with acute HEV infection. Overall, intra-host HEV diversity was low when assessed at a single time point, consistent with a narrow transmission bottleneck and strong early immune-mediated clearance. Nonetheless, several mutations that showed severely impaired replication *in vitro* were repeatedly detected across donors. Their replication defects could be rescued in a polymerase *trans*-complementation system, suggesting that transient co-existence with replication-competent genomes may allow otherwise deleterious variants to exist *in vivo*. In donors with longitudinal samples, we observed temporal shifts in the frequency of individual substitutions, underscoring the value of time-resolved sampling to capture early selection dynamics.

The subtype distribution in our cohort was consistent with prior epidemiological reports from Central Europe, with HEV-3c dominating (32). Although closely related, HEV-3 subtypes showed characteristic polymerase substitutions, mirroring earlier suggestions of subtype-specific influences on viral titers, disease severity, and clinical outcome (32–35). While our data set reveals subtype-specific genetic differences within the HEV-3 polymerase, the cohort size precludes the identification of robust markers associated with clinical outcomes. Interestingly, we detected substitutions in treatment-naïve individuals that are identical to those previously reported to emerge during antiviral therapy. Rather than indicating a predisposition to progression into chronic infection or antiviral resistance, the presence of these variants in acute, asymptomatic donors likely reflects the natural landscape of the HEV sequence spectrum. This interpretation is supported by their occurrence in wastewater samples (36). Whether specific genetic markers increase the likelihood of chronic infection remains unresolved and cannot be addressed within the scope of our data set. Resolving this question will require longitudinal surveillance from early acute infection through to chronicity, complemented by mechanistic studies *in vitro* to assess the contribution of individual substitutions to viral fitness and persistence. The relationship between intra-host diversity and adaptive capacity has been well documented in other RNA viruses. For instance, increased genetic diversity in poliovirus facilitates adaptation to varied cellular environments (37), and a high degree of diversification during acute HCV infection has been linked to progression to chronic infection (17). Although HEV is usually self-limiting in immunocompetent hosts, diversification early in acute infection may still be important. Greater genetic diversity could, for instance, prolong infection and viral shedding or enable HEV to establish infection in multiple organs, a possibility supported by clinical reports of extrahepatic disease (38, 39) and *in vitro* evidence of replication in diverse cell types (40–45). In chronically infected patients, higher diversity during the initial phase of infection could potentially promote persistence, as with HCV; however, for HEV, this remains a hypothesis that warrants investigation in future studies. Importantly, comparisons between immunocompetent acute infection and chronic infection in immunocompromised patients must consider the fundamentally different immune environments. Reduced immune pressure in immunocompromised patients with chronic infection may enable prolonged replication and greater evolutionary breadth. Consistent with this, chronic HEV patients in our data set displayed markedly higher numbers of SNVs and amino-acid substitutions, whereas high-frequency variants were nearly absent in donors with acute infection. This pattern is consistent with previous studies reporting increased nucleotide heterogeneity in chronic infection across multiple genomic regions, including ORF2, the proline-rich region (PPR), and the macrodomain (46, 47). By extending these observations to the RdRp, our findings further support the concept that chronic infection provides an expanded evolutionary landscape. Incorporating samples from a previous data set (11) similarly underscores the substantially higher evolutionary capacity during chronic infection compared to the constrained diversity observed during acute infection.

Despite the lower diversity observed during acute vs chronic infection, a distinct cluster of four mutations was observed in a subset of acutely infected donors, including a substitution affecting the conserved GDD catalytic motif, which is absent from available GenBank HEV sequences. Interestingly, one longitudinally enriched substitution, L1524F, lies within the described HLA-A*02:01-restricted epitope LLWNTVWNM (48, 49). Another observed mutation occurred only one residue away from an additional previously reported epitope. While speculative at this stage, the proximity of these substitutions to known CD8^+^ T-cell epitopes raises the possibility of early immune-driven selection in some donors. Further work involving epitope mapping and functional immune assays will be needed to determine whether such variants modulate antigen presentation or T-cell recognition. Although most of the selected substitutions were deleterious *in vitro*, one variant replicated close to wild-type levels, and the others could be rescued by polymerase *trans*-complementation. Notably, the trans-complementation system showed only a modest increase in replication, likely reflecting competition between co-transfected genomes for limiting host factors. Together with their repeated detection *in vivo*, these findings argue against sequencing artifacts and suggest that variants with decreased replicative fitness may exist within mixed viral populations.

Additionally, premature stop codons were identified in several acute-phase donors. These genomes, which likely encode truncated or non-functional proteins, were rare or absent in chronic infection, both with and without ribavirin treatment. Defective viral genomes of this kind have been extensively documented in other RNA viruses, where they exert a profound influence on replication dynamics, immune recognition, and population structure (50–52). In the context of HEV, defective ORF2 variants have been associated with the formation of decoy antigens and immune modulation (53). To assess whether similar patterns of genetic heterogeneity occur outside the RdRp, we additionally analyzed the overlapping ORF2/ORF3 region. Overall, diversity patterns in this region were comparable to those observed in the RdRp, consistent with previous reports (11). Notably, the sequenced ORF2 fragment corresponds to the N-terminal part of the capsid protein, encompassing the signal peptide and parts of the shell domain while excluding the major antigenic region (54). A substitution within this region, P79S, has previously been linked to the generation of immunogenic decoys; however, this specific variant was not detected in our cohort. Instead, we identified other previously described substitutions, P25S and A64T, which have been reported to exert a neutral effect on viral infectivity *in vitro* (53). In addition to neutral substitutions within ORF2, we identified RdRp mutations potentially representing defective viral genomes, including premature stop codons in several acute-phase donors. The prevalence of defective genomes and low-frequency substitutions during the acute phase suggests that such variants emerge frequently during the initial stages of viral replication, possibly reflecting replication-associated noise and transient stochastic exploration of sequence space during periods of high viral replication. However, as viral populations mature and stabilize during prolonged infection, defective or deleterious genomes are likely outcompeted and progressively purged from the viral population. Serial samples from four donors provided rare insight into short-term viral evolution during asymptomatic acute HEV infection. Even though overall diversity remained low, individual substitutions emerged or increased in frequency over short intervals, consistent with early selection or stochastic expansion of minority variants. Such dynamics would be missed by cross-sectional sampling alone.

In summary, the findings demonstrate that HEV evolution during the acute phase of infection in immunocompetent hosts is strongly constrained, shaped by narrow transmission bottlenecks and effective immune clearance. However, under conditions such as mixed infection, broader bottlenecks, or localized immune pressures, greater early intra-host diversity can emerge. It is noteworthy that several substitutions observed in acute donors were deleterious *in vitro*. However, these substitutions could be rescued through polymerase trans-complementation. This finding indicates that transient co-existence with replication-competent genomes may allow impaired variants to exist independently of the specific mutational sites involved. Conversely, chronic infection in immunosuppressed hosts can permit extensive diversification, thereby fostering the emergence and maintenance of adaptive variants. A more profound comprehension of these intra-host dynamics is pivotal for predicting viral evolution, optimizing therapeutic strategies, and evaluating the clinical risks posed by circulating HEV variants.

## Data Availability

Associated NGS data are available at the SRA (PRJNA1478978).

## SUPPLEMENTAL MATERIAL

Supplemental figures mbio.01151-26-s0001.docx Figures S1 to S12.

ASM does not own the copyrights to Supplemental Material that may be linked to, or accessed through, an article. The authors have granted ASM a non-exclusive, world-wide license to publish the Supplemental Material files. Please contact the corresponding author directly for reuse.
