# Supplementary material for: Intra-host viral population dynamics during acute hepatitis E virus infection
Source: mBio. 2026 Jul 10;17(8):e01151-26. doi: 10.1128/mbio.01151-26 (PMC13463781; doi:10.1128/mbio.01151-26)
Supplement: Supplementary file 1 [file mbio.01151-26-s0001.docx]

**Intra-host viral population dynamics during acute hepatitis E virus infection**

Saskia Janshoff^1,2,3,4^, Ricarda Plümers^3^, Alina Kohl^1, 4^, Maximilian Klaus Nocke^2,4,5^, Patrick Behrendt^6,7,8^, Cornelius Knabbe^9^, Rui Costa^1, 4^, Tanja Vollmer^3^, Daniel Todt^2,4,5*^, Eike Steinmann^1,4*^, André Gömer^1,4*^

1 Department of Molecular and Medical Virology, Ruhr University Bochum, Bochum, Germany

2 Department of Translational and Computational Infection Research (TRACiR), Ruhr University Bochum, Bochum, Germany

3 Institut für Laboratoriums- und Transfusionsmedizin, Herz- und Diabeteszentrum Nordrhein-Westfalen, Universitätsklinikum der Ruhr-Universität Bochum, Bad Oeynhausen, Germany

4 Hepatitis E Virus Research Hub (HepE-Hub), Bochum, Germany

5 European Virus Bioinformatics Center (EVBC), Jena, Germany

6 TWINCORE, Centre for Experimental and Clinical Infection Research, a joint venture between Helmholtz Centre for Infection Research and Hannover Medical School, Hannover, Germany.

7 Department of Gastroenterology, Hepatology, Infectious Diseases and Endocrinology, Hannover Medical School, Hannover, Germany.

8 German Center for Infection Research (DZIF), Braunschweig, Germany.

9 Institut für Laboratoriums- und Transfusionsmedizin, Herz- und Diabeteszentrum Nordrhein-Westfalen, Universität Bielefeld, Bad Oeynhausen, Germany

* shared contribution

Figure S1: Distribution of HEV-3 subtypes and mutation profile in acutely infected donors. (A) Subtype distribution among blood donors (%). (B) The number of variants per polymerase domain at the nucleotide (blue) and amino acid (orange) levels.

Figure S2. Coverage distribution across all samples.

Figure S3: Coverage profiles of two samples excluded from the analysis due to low overall coverage.

Figure S4: Distribution of nucleotide variants per sample. The lower dotted line indicates the error threshold (3.287%), and the upper dotted line marks the threshold for high-frequency variants (20%).

Figure S5: Shannon entropy distribution for each donor.

Figure S6: (A) Variant distribution across all donors.

Figure S7: Entropy comparison between chronic HEV (CHE) patients and acutely infected donors (black). CHE patients not treated with antivirals are shown in blue, and patients treated with ribavirin (RBV) are shown in orange.

Figure S8: Amino acid variant distribution per donor.

Figure S9: Nucleotide and amino acid variant distribution in 13 matched donors across ORF1 and ORF2/3 sequences. (A, B) Nucleotide variant frequency along the analyzed amplicons. The lower dotted line indicates the error threshold (3.287%), and the upper dotted line marks the threshold for high-frequency variants (20%). Bars at the top indicate the genomic regions encoding ORF1, ORF2, and ORF3 proteins. (C) Quantification of nucleotide variants and (D) their frequency density distribution. (E) Quantification of amino acid substitutions within each ORF and (F) their frequency density distribution.

Figure S10: Heatmap of selected amino acid substitutions. Shown are substitutions detected at frequencies above 10% in at least one patient. Colors indicate substitution frequency, ranging from low frequency (blue) to intermediate frequency (yellow) and high frequency (red).

Figure S11: Mutation location on an alpha-fold generated 3D structure prediction. Red indicates C-terminal and N-terminus of the RdRp. Substitutions that were enriched in acutely infected individuals are highlighted in yellow.

Figure S12: Cumulative stop codon frequency across cohorts. Acutely infected donors are shown in pink, while untreated CHE patients are shown in blue and RBV-treated CHE patients in orange.
